# Clinical characteristics, prognosis, and predictive modeling in class IV ± V lupus nephritis

**DOI:** 10.3389/fimmu.2025.1580146

**Published:** 2025-05-15

**Authors:** Anjing Wang, Yunlong Qin, Yan Xing, Zixian Yu, Liuyifei Huang, Jinguo Yuan, Yueqing Hui, Mei Han, Guoshuang Xu, Jin Zhao, Shiren Sun

**Affiliations:** ^1^ Department of Nephrology, Xijing Hospital, The Fourth Military Medical University, Xi’an, Shaanxi, China; ^2^ Department of Postgraduate Student, Xi’an Medical University, Xi’an, Shaanxi, China; ^3^ Department of Nephrology, Bethune International Peace Hospital, Shijiazhuang, China

**Keywords:** lupus nephritis, class IV ± V, machine learning, risk factors, prognostic model

## Abstract

**Objective:**

The objective of this study is to compare the clinical features and survival outcomes of class IV ± V lupus nephritis (LN) patients, identify risk factors, and develop an accurate prognostic model.

**Methods:**

This study enrolled patients diagnosed with class IV ± V LN by renal biopsy at Xijing Hospital from December 2013 to June 2023. The composite endpoint of the study was defined as a decline in the estimated glomerular filtration rate (eGFR) by more than 50%, progression to end stage renal disease, or death, whichever came first. The eGFR was calculated utilizing the Chronic Kidney Disease Epidemiology Collaboration (CKD-EPI) formula. ESRD is defined as an eGFR less than 15ml/min/1.73m^2^, necessitating the commencement of chronic dialysis (hemodialysis or peritoneal dialysis) or kidney transplantation. We compared the baseline features and survival prognosis between patients with class IV ± V LN. The prognostic model was developed using machine learning algorithms and Cox regression. The model’s performance was evaluated in terms of discrimination, calibration, and risk classification using the concordance index (C-index), integrated brier score (IBS), net reclassification index (NRI), and integrated discrimination improvement (IDI), respectively.

**Results:**

A total of 313 patients were enrolled for this study, including 156 class IV and 157 class IV+V LN. During the median follow-up period of 42.6 (17.0, 83.4) months, 35 (22.4%) class IV and 38 (24.2%) class IV+V LN patients experienced combined events. Class IV and class IV+V patients have similar clinical manifestations, treatment strategies, and long-term prognosis, despite class IV having a higher chronic index (CI) score (*P* < 0.001). Seven eligible variables (eGFR, CI, age, basophil percentage, red blood cell count, mean arterial blood pressure, and uric acid) were selected to develop the random survival forest (RSF) model. This model demonstrated the best performance with a C-index of 0.771 (0.667, 0.848) and an IBS of 0.144 (0.132, 0.154). The IDI and NRI in the testing set further confirmed that the RSF model exhibited superior risk classification and discrimination capabilities.

**Conclusion:**

Class IV ± V LN was similar in clinical manifestations, treatment strategies, and long-term prognosis, despite differences in pathological features. The RSF model we established for class IV ± V LN patients, incorporating seven risk factors, exhibits superior survival prediction and provides more precise prognostic stratification.

## 1 Introduction

Systemic Lupus Erythematosus (SLE) is a complicated autoimmune disease characterized by its multisystem involvement and diverse clinical manifestations (1, 2). Lupus nephritis (LN) is one of the most severe organ manifestations of SLE (3). The incidence of LN among adult SLE patients ranges from approximately 30% to 60% (3, 4). The pathogenesis of LN is primarily manifested through the deposition of autoantibodies and immune complexes, activation and/or proliferation of infiltrating immune cells, as well as kidney resident cells (4). The presence of LN can significantly increase SLE-associated mortality and morbidity (5). Between 5 and 30% of patients progress to end-stage renal disease (ESRD) within a decade following the initial diagnosis of LN (6). Despite the ongoing development of immunomodulatory agents and supportive care, the prognosis of LN has not seen substantial improvement over the past 10 years (7).

The renal pathological features of LN play a crucial role in guiding therapeutic strategies and providing prognostic information (8). The 2003 International Society of Nephrology/Renal Pathology Society (ISN/RPS) classification system categorizes LN into six pathological types, which has been widely accepted (9, 10). Notably, class V may present simultaneously with class III or IV, known as class III/IV+V, and these are associated with severe symptoms that require intensive therapy (11). Among these, patients with class IV ± V LN tend to have the worst renal outcomes, but the prognosis and clinical features between class IV and class IV+V are inconsistent across various studies (12–14). However, the treatment guidelines for the treatment of class IV ± V patients are similar (15, 16). Therefore, it is necessary to compare the clinical features and further evaluate the prognosis of class IV ± V. Various investigations have explored clinical and serological markers such as serum creatinine levels, proteinuria levels, haematuria, and hypertension as predictors of the prognosis of LN (17–20). Unfortunately, there are limited retrospective studies that focus solely on the prognosis of class IV ± V, which leads to an incomplete understanding of the risk factors influencing disease progression and a lack of comprehensive clinical guidance for the prognosis of class IV ± V.

Machine Learning (ML) is currently receiving increasing attention in clinical prediction modelling (21). The random survival forest (RSF) is a ML algorithm specifically designed for predicting survival outcomes (22). eXtreme Gradient boosting (XGboost) has gained widespread recognition in numerous ML and data mining challenges (23). However, ML has not yet been applied to the prediction of long-term prognosis of class IV ± V LN patients. Therefore, this study aims to compare the clinical features and prognosis, explore the risk factors impacting the long-term prognosis of class IV ± V LN patients diagnosed through renal biopsy, and establish an accurate prognostic model by employing ML algorithms, which providing valuable insights for early disease intervention and aiding physicians in decision-making.

## 2 Materials and methods

### 2.1 Study population

We gathered the clinical and pathological information of patients who underwent renal biopsy and were subsequently diagnosed with class IV ± V LN at Xijing Hospital of The Fourth Military Medical University from December 2013 to June 2023. The inclusion criteria encompassed the following: (1) age 18 years or older; (2) a diagnosis of class IV ± V LN confirmed by renal biopsy with complete follow-up records. The exclusion criteria encompassed the following: (1) patients with complications of other secondary nephropathies, such as diabetic nephropathy; (2) biopsy specimens with less than 8 glomeruli detected or absence of original pathological reports; (3) presence of other serious diseases with a life expectancy of less than 1 year; (4) unavailable outcome data. This study complied with the reporting guidelines for the Transparent Reporting of a Multivariable Prediction Model for Individual Prognosis or Diagnosis (TRIPOD) (24) (**Supplementary Table S1**). The retrospective design of this study made patient informed consent unnecessary, as approved by the Ethics Committee of Xijing Hospital (ethical number: KY20213027-1).

### 2.2 Outcomes

In this study, the endpoint was the composite outcome of decline in estimated glomerular filtration rate (eGFR) of more than 50%, ESRD, or death, whichever came first. The eGFR was calculated utilizing the Chronic Kidney Disease Epidemiology Collaboration (CKD-EPI) formula (25). ESRD is defined as an eGFR less than 15ml/min/1.73m^2^, necessitating the commencement of chronic dialysis (hemodialysis or peritoneal dialysis) or kidney transplantation (26). Follow-up duration was the time interval between renal biopsy and the final outpatient visit or telephone follow-up.

### 2.3 Data collection

Two researchers (Wang and Zhao) collected the demographic, clinical, and pathological data at baseline from the electronic medical record information system (**Supplementary Table S2**). We compared the baseline data between the class IV and IV+V LN patients (**Table 1**). The treatment methods employed in this study were categorized into four types: (1) corticosteroids monotherapy, (2) combination therapy with corticosteroids and immunosuppressants, (3) combination therapy with corticosteroids, immunosuppressants, and novel biological agents, and (4) other treatments. Comorbidity or other diseases were documented based on the diagnostic results of patients. Patients with a follow-up duration of at least 6 months were included in the analysis, unless they reached the predefined endpoints. During follow-up, we evaluated the survival status of patients, progression to ESRD, initiation of dialysis, and laboratory examination data. Rigorous safeguards were implemented to guarantee the privacy of patients’ information throughout the data collection process and all subsequent stages.

**Table 1:** Comparison of baseline characteristics between class IV and class IV+V.

Characteristics	Class IV ± V (n=313)	Class IV (n=156)	Class IV+V (n=157)	*P-value*
Baseline (at renal biopsy)				
Age (years)	35.4 ± 13.3	36.3 ± 14.0	34.5 ± 12.7	0.277
Female	265 (84.7%)	132 (84.6%)	133 (84.7%)	0.981
^a^BMI (kg/m^2^)	23.00 ± 3.84	22.52 ± 3.61	23.40 ± 4.02	**0.043**
Duration of SLE (months)	6.0 (2.0, 48.0)	3.5 (1.0, 29.8)	12 (2, 54)	**0.001**
^b^MAP (mmHg)	100 (88, 115)	98 (87, 115)	104 (91, 115)	0.050
UTP (mg/24h)	2100 (1090, 4030)	1980 (872, 4315)	2090 (1254, 3993)	0.160
WBC (×10^9^/L)	5.18 (3.51, 7.09)	4.70 (3.40, 6.91)	5.39 (3.82, 7.27)	0.220
BASO%	0.209 ± 0.261	0.228 ± 0.232	0.190 ± 0.286	**0.025**
RBC (×10^9^/L)	3.53 ± 0.76	3.43 ± 0.72	3.63 ± 0.78	**0.020**
Hb (g/L)	103.0	101.1 ± 21.3	104.5 ± 0.3	**0.049**
PLT (×10^9^/L)	152 (104, 202)	144 (89, 193)	155 (113,210)	0.069
Urinary RBC (μL)	174.0 (49.8, 565.0)	188.7 (50.4, 610.6)	153.4 (42.5, 491.5)	0.308
Urinary CAST (uL)	2.12 (1.06, 10.80)	2.29 (1.05, 11.26)	2.00 (1.08, 9.97)	0.803
Urinary BACT (uL)	223.8 (70.4, 686.7)	204.0 (70.8, 748.8)	252.9 (57.1, 655.8)	0.912
Urinary Path CAST (uL)	0.98 (0.53, 4.57)	1.00 (0.47, 5.54)	0.95 (0.56, 3.75)	0.943
BUN (mmol/l)	8.35 (5.32, 12.60)	8.25 (5.55, 12.66)	8.39 (5.17, 12.48)	0.889
^c^eGFR (ml/min/1.73m^2^)	67.5 (43.1, 89.2)	67.1 (45.4, 89.2)	67.6 (50.0, 91.3)	0.921
Serum creatinine (μmol/L)	96.0 (77.0, 141.0)	97.0 (77.3, 141.8)	96.0 (76.0, 141.0)	0.976
UA (μmol/l)	383 (304, 461)	384 (303, 463)	379 (304, 463)	0.896
Serum albumin (g/L)	27.2 ± 6.9	28.6 ± 6.6	26.0 ± 6.9	**< 0.001**
Serum IgG (g/L)	11.0 (15.8, 7.3)	11.5 (7.8, 16.2)	11.0 (6.47, 15.8)	0.212
Serum IgA (g/L)	2.39 (1.81, 3.21)	2.52 (1.89, 3.34)	2.28 (1.67, 3.11)	0.122
Serum IgM (g/L)	0.89 (0.61, 1.33)	0.90 (0.64, 1.35)	0.89 (0.59, 1.31)	0.374
Serum tIgE (g/L)	109 (27.3, 361.5)	116.5 (29.7, 354.3)	78.2 (27.5, 377.0)	0.506
Serum C3 (mg/dl)	0.40 (0.28, 0.56)	0.38 (0.27, 0.57)	0.41 (0.29, 0.56)	0.394
Serum C4 (mg/dl)	0.07 (0.04, 0.12)	0.06 (0.04, 0.11)	0.07 (0.04, 0.12)	0.092
hs.CRP (mg/dl)	1.93 (0.81, 6.56)	1.54 (0.81, 5.74)	2.85 (0.83, 8.96)	0.078
Positive ANA	308 (98.4%)	153 (98.1%)	155 (98.7%)	0.721
Positive anti-dsDNA	128 (40.9%)	70 (44.9%)	58 (36.9%)	0.154
AI	7.00 (5.00, 9.00)	6.00 (5.00, 9.00)	7.00 (5.00, 9.00)	0.104
CI	2.00 (1.00, 3.00)	1.00 (1.00, 3.00)	2.00 (1.00, 3.00)	**< 0.001**
Extrarenal manifestations				
Fever	71 (22.7%)	34 (21.8%)	37 (23.6%)	0.708
Arthralgia	92 (29.4%)	46 (29.5%)	46 (29.3%)	0.971
Photosensitivity	30 (9.6%)	11 (7.1%)	19 (12.1%)	0.129
Rash	30 (9.6%)	14 (9%)	16 (10.2%)	0.715
Alopecia	95 (30.4%)	41 (26.3%)	54 (34.4%)	0.119
Oral ulcers	26 (8.3%)	9 (5.8%)	18 (11.4%)	0.073
Hypertension	82 (26.2%)	53 (34.0%)	29 (18.5%)	**0.002**
Induction therapy				
Glucocorticoid only	19 (6.1%)	6 (3.8%)	13 (8.3%)	0.100
Mycophenolate mofetil	75 (24%)	41 (26.3%)	34 (21.7%)	0.338
Cyclophosphamide	179 (57.2%)	92 (59%)	87 (55.4%)	0.524
Tacrolimus	10 (3.2%)	5 (3.2%)	5 (3.2%)	0.991
Novel biological agents	12 (3.9%)	2 (1.3%)	10 (6.4%)	0.019
Follow-up parameters				
Follow-up (months)	42.6 (17.0, 83.4)	39.1 (18.7, 71.0)	48.5 (13.1, 98.1)	0.059
Endpoint	73 (23.3%)	35 (22.4%)	38 (24.2%)	0.712
≥50% loss of eGFR	21 (6.7%)	9 (5.8%)	12 (7.6%)	0.652
ESRD	26 (8.3%)	12 (7.7%)	14 (8.9%)	0.838
Death	26 (8.3%)	14 (9.0%)	12 (7.6%)	0.688

Values are presented as the mean ± standard deviation, median (interquartile range) or n (%). LN, lupus nephritis; SLE, systemic lupus erythematosus; MAP, mean arterial pressure; BMI, body mass index; UTP, urine total protein; WBC, white blood cell; BASO%, basophil percentage; RBC, red blood cell; Hb, hemoglobin; HCT, haematocrit; PLT, platelet; BACT, bacteria CAST; Path, pathological; BUN, blood urea nitrogen; eGFR, estimated glomerular filtration rate; UA, uric acid; hs.CRP, hypersensitive C-reactive protein; AI, activity index; CI, chronicity index; ANA, antinuclear antibodies; anti-dsDNA, anti-double stranded deoxyribonucleic antibody; anti-Sm, anti-Smith antibody; anti-RNP, anti-ribosomal P antibody; anti-SSA, anti-SSA antibody; anti-SSB, anti-SSB antibody.

a BMI was calculated as weight/height^2^;

b MAP was calculated by diastolic blood pressure + systolic blood pressure/3 based on initial blood pressure measurements upon admission;

c eGFR was calculated by the CKD-EPI (Chronic Kidney Disease Epidemiology Collaboration) equation. The bold values represents the characteristics with P < 0.05.

### 2.4 Renal pathological assessment

Experienced pathologists at this center conducted a review of the pathological characteristics of LN. The pathological index of LN refers to the activity index (AI) and chronic index (CI) scoring system of the National Institutes of Health (NIH) (27). The AI score comprises six components: endocapillary hypercellularity, fibrinoid necrosis, cellular/fibrocellular crescents, neutrophils/karyorrhexis, interstitial inflammation, and hyaline deposits. The CI score consists of four components: interstitial fibrosis, fibrous crescents, total glomerulosclerosis score, and tubular atrophy. Each component is assigned a score of 0 to 3 based on the percentage of glomeruli or the affected cortex area. It is noteworthy that cellular/fibrocellular crescents and fibrinoid necrosis are given double weight. By summing the scores of the individual components, we obtained a cumulative AI score of 24 and a CI score of 12, respectively.

### 2.5 Model development and performance

Model development and internal validation were conducted using R software (Version 4.3.2).The primary basis for variables selection was variable importance (VIMP), an internal statistic of the RSF algorithm (28). Bootstrapping resampling with 1000 replications was conducted, and the 95% confidence interval (CI) of VIMP was calculated. Variables with a mean VIMP exceeding 0.02 were considered as RSF prognostic variables. Additionally, univariate and multivariate Cox regression analyses were employed for variable selection. The final selection of variables was determined by integrating the results of these two methods with clinical experience. The dataset was randomly divided into a training set and a testing set at a ratio of 6:4. The optimal parameters for each model were chosen through the method of grid search. For the RSF model, the parameters were set as follows: the ntree to 800, the mtry to 2, and the nodesize to 3 (**Supplementary Figure S1**). For the XGboost model, For the XGboost model, the parameters were: learning rate at 0.01, tree depth at 1, subsample at 1, colsample_bytree at 1, and gamma at 0.5. The specific R packages utilized were “survival”, “MASS” “randomForestSRC”, “survivalsvm”, and “XGboost”.

The discriminative capacity of the model was evaluated using the area under the receiver operating characteristic curve (AUC) and the concordance index (C-index). To assess the model’s discriminative capacity throughout the entire study duration, we computed the area under the time-dependent curve (tAUC). The integrated brier score (IBS) was calculated and Calibration plots were generated to estimate the calibration ability of ML models. Additionally, the decision curve analysis (DCA) was performed to further assess model performance. The integrated discrimination improvement (IDI) and net reclassification improvement (NRI) were also determined to evaluate the risk reclassification. A higher NRI value indicates that the newly established models demonstrated superior performance compared to the Cox model in reducing the risk of misclassifying individuals. Meanwhile, a higher IDI value suggests a more significant improvement in the predictive accuracy of the newly established models.

### 2.6 Statistical analysis

Variables with missing data rates above 20% and with a correlation coefficient surpassing 0.75 were eliminated from the analysis. Random forest imputation was utilized to compensate for missing data. Categorical variables were reported as frequencies and percentages, and comparisons were made using the χ^2^ test or Fisher’s exact test, as appropriate. Continuous variables that were normally distributed were reported as mean ± standard deviation (SD), while those with non-normal distributions were reported as median (interquartile ranges). Comparisons between groups were performed utilizing the Mann-Whitney U test or Student’s t-test. Optimal cut-off points for continuous variables were determined using the Jsurvival-Survival Module in the ClinicoPath software suite in Jamovi, which was used to identify significant correlations with survival outcomes (29). Kaplan-Meier curves were adopted for cumulative survival analysis and group comparisons were made via the log-rank test. The two-tailed test was utilized to calculated *P* values, and a *P*-value less than 0.05 was designated as indicative of statistical significance. All statistical analyses were executed in R (Version 4.3.2), SPSS (IBM Version 27.0), and Jamovi (Version 2.3, Sydney, Australia).

## 3 Results

### 3.1 Patient characteristics

**Figure 1:**
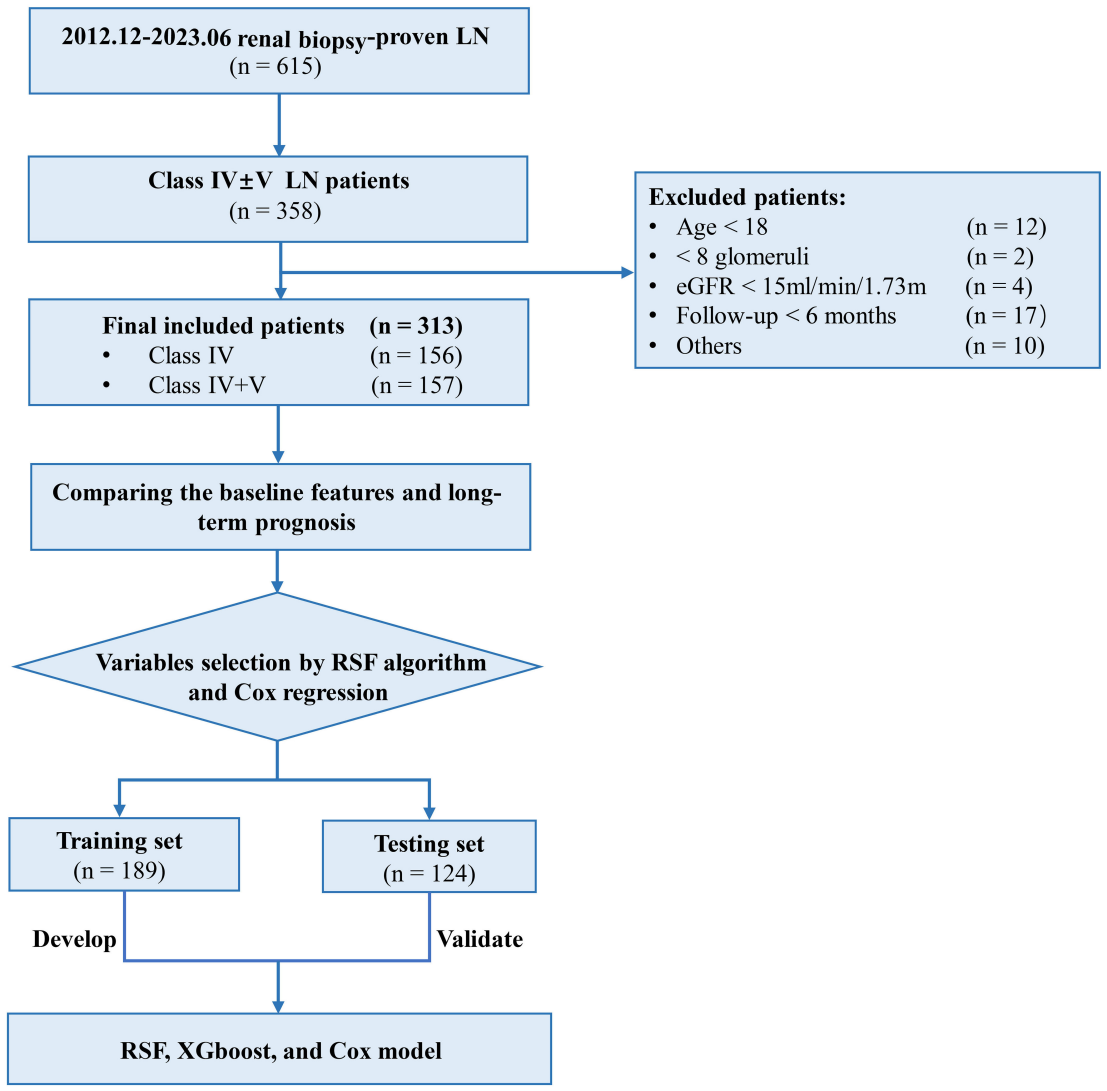
Study flowchart. LN, lupus nephritis; eGFR, estimated glomerular filtration rate; RSF, random survival forest; XGboost, eXtreme Gradient Boosting.

A total of 313 eligible class IV ± V LN patients were included from a pool of 615 biopsy-proven LN patients, consisting of 156 with class IV and 157 with class IV+V LN (**Figure 1**). During the median follow-up duration of 42.6 (17.0, 83.4) months, 35 (22.4%) class IV and 38 (24.2%) LN patients experienced combined events with no statistically significant (*P* = 0.712). In detail, 9 (5.8%) class IV and 38 (24.2%) LN patients experienced a decline of eGFR more than 50%, 12 (7.7%) class IV and 14 (8.9%) LN patients progress to ESRD, and 14 (9.0%) class IV and 12 (7.6%) LN patients occurred death. Between the two group, the three events were all with no statistically significant (*P* = 0.652, *P* = 0.838, *P* = 0.688). The platelet was 152 (104, 202) × 10^9^/L, the white blood cell was 5.18 (3.51, 7.09) × 10^9^/L, the hypersensitive C-reactive protein was 1.93 (0.81, 6.54) mg/L, the MAP was 100 (88.0, 115) mmHg, the urinary total protein (UTP) was 2100 (1090, 4030) mg/24h, and the eGFR was 67.5 (43.1, 89.2) mL/min/1.73 m^2^. The AI score was 7.00 (5.00, 9.00) and CI score was 2.00 (1.00, 3.00) (**Table 1**).

### 3.2 Comparisons of the baseline data and prognosis of class IV ± V LN patients

Class IV and class IV+V LN exhibited similar clinical manifestations in terms of the UTP (*P* = 0.160), eGFR (*P* = 0.921), urinary red blood cell (RBC) (*P* = 0.308), and anti-dsDNA positivity rate (*P* = 0.154) and similar initial therapeutic regimen primarily including glucocorticoid combined with mycophenolate mofetil (*P* = 0.338) or cyclophosphamide (*P* = 0.991). Regarding the pathological features, there was no significant difference in the AI score between the two groups (*P* = 0.104), although the CI score (*P* < 0.001) was significant higher in class IV+V. Laboratory examinations revealed that class IV LN patients have higher levels of albumin (*P* = 0.049) and basophil percentage (BASO%) (*P* = 0.025) and lower levels of RBC (*P* = 0.020) and hemoglobin (*P* = 0.049). Kaplan-Meier survival curve analysis revealed no significant difference in renal survival rates between class IV and class IV+V LN patients (*P* = 0.710) (**Supplementary Figure S2**).

### 3.3 Screening of variables

**Figure 2:**
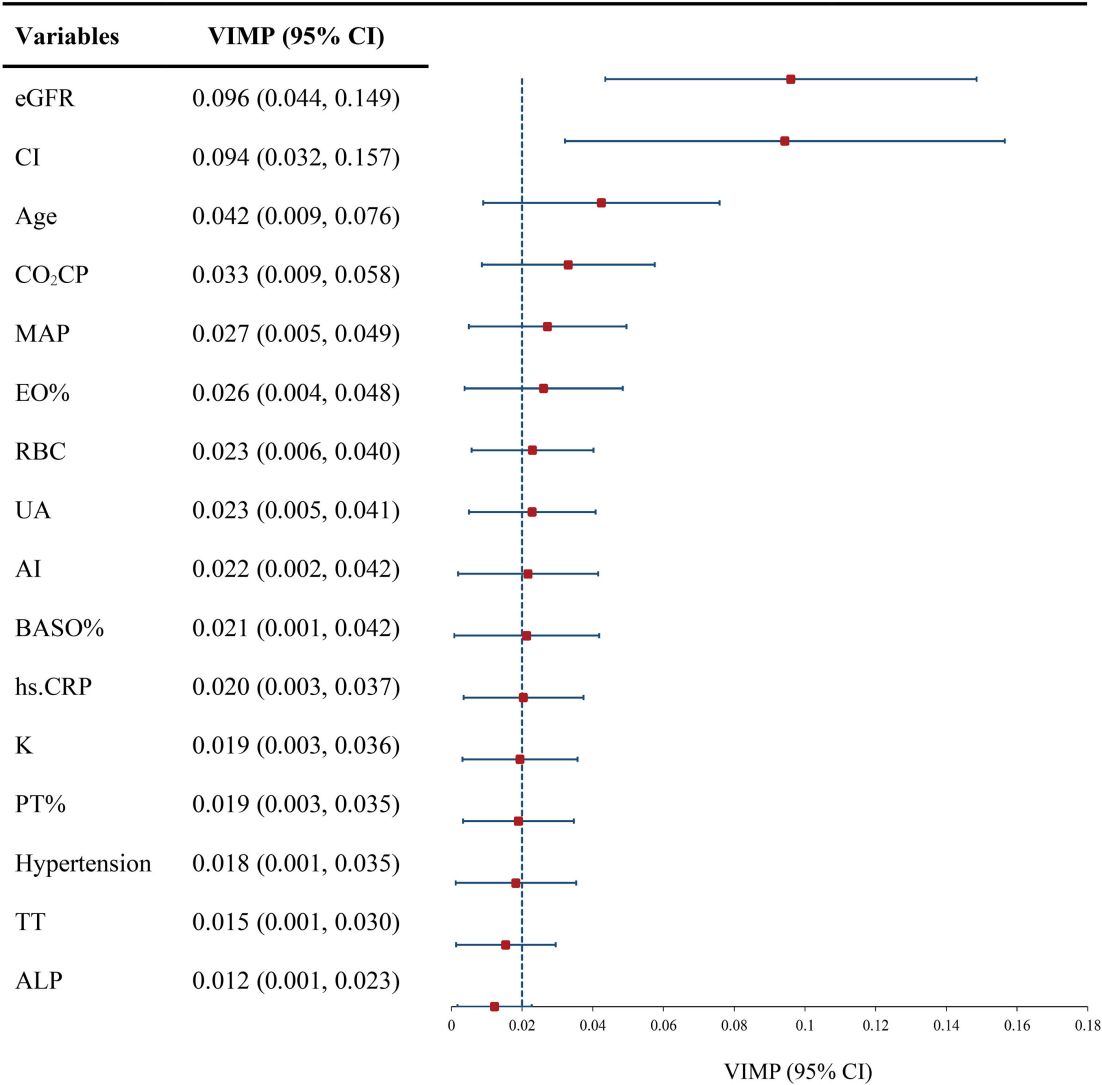
VIMP of indicators for developing model. eGFR, estimated glomerular filtration rate; CI, chronic index; MAP, mean arterial blood pressure; EO%, eosinophil percentage; RBC, red blood cell; UA, uric acid; AI, activity index; BASO%, basophil percentage; hs.CRP, hypersensitive C-reactive protein; PT%, prothrombin time percentage; TT, thrombin time; ALP, alkaline phosphatase; VIMP, variable importance; Dotted line, mean VIMP.

After applying the RSF algorithm for data imputation and eliminating collinear data, 99 covariates were chosen from an initial pool of 114 variables for subsequent analysis. 11 important prognostic variables were filtered out as RSF prognostic variables based on VIMP scores (**Supplementary Table S3**). In addition, multifactor Cox regression analysis identified age, eGFR, uric acid (UA), BASO%, RBC, CI, and prothrombin activity ratio as independent risk factors impacting the outcome of class IV ± V LN patients. Combining these findings with clinical experience, seven prognostic variables were determined eGFR, CI, age, BASO%, RBC, MAP, and UA based on the VIMP value sorting. These variables were then integrated into the final model as the ultimate variables (**Figure 2**).

### 3.4 Model development and performance

#### 3.4.1 Comparison of the discriminatory capabilities of RSF, XGboost, and Cox model

All patients were randomly divided into 189 training samples and 124 testing samples (**Table 2**). The clinical and pathological variables as well as the follow-up data were well-balanced between the training and testing cohorts, indicating a robust study design. After 12 months, tAUC was consistently higher compare to the RSF model than in the Cox model and XGboost model in both the training and testing cohort.

**Table 2:** Comparison of baseline and follow-up characteristics between the training and testing cohorts.

Characteristics	Training cohort N=189	Testing cohort N=124	*P-value*
Baseline (at renal biopsy)			
Age (years)	34.8 ± 12.9	36.4 ± 14.1	0.302
Female	162 (85.7%)	103 (83.1%)	0.634
LN classification			
Class IV	91 (48.1%)	65 (52.4%)	
Class IV + V	98 (51.9%)	59 (47.6%)	
Duration of SLE (months)	6.00 (1.5, 48.0)	6.00 (2.0, 48.0)	0.580
^a^BMI (kg/m^2^)	23.10 ± 3.96	22.70 ± 3.66	0.599
^b^MAP (mmHg)	102 (89, 116)	98.0 (87, 111)	0.122
UTP (mg/24h)	2205 (1008, 4480)	2039 (1102, 3646)	0.883
WBC (×10^9^/L)	5.20 (3.73, 7.05)	5.12 (3.41, 7.15)	0.757
BASO%	0.211 ± 0.268	0.206 ± 0.250	0.590
RBC (×10^9^/L)	3.55 ± 0.76	3.49 ± 0.76	0.522
Hb (g/L)	102.0 ± 21.9	103.0 ± 22.8	0.732
PLT (×10^9^/L)	155 (102, 210)	144 (104, 193)	0.572
Urinary RBC (μL)	174 (40.3, 590.0)	175 (77.6, 494.0)	0.314
Urinary CAST (uL)	2.00 (1.05, 8.54)	2.30 (1.10, 15.10)	0.146
BUN (mmol/l)	8.30 (5.50, 13.10)	8.41 (5.18, 11.20)	0.351
^c^eGFR (ml/min/1.73m^2^)	66.2 (40.6, 88.9)	69.0 (47.8, 91.2)	0.440
Serum creatinine (μmol/L)	98.0 (77.0, 144.0)	94.5 (76.8, 132.0)	0.457
UA (μmol/l)	385 (304, 466)	371 (303, 452)	0.471
CysC (mg/L)	1.77 (1.30, 2.63)	1.69 (1.34, 2.26)	0.374
Serum albumin (g/L)	26.80 ± 6.87	27.80 ± 6.81	0.193
C3 (mg/dl)	0.39 (0.28, 0.56)	0.42 (0.28, 0.56)	0.509
C4 (mg/dl)	0.23 ± 1.22	0.10 ± 0.22	0.168
hs.CRP (mg/L)	2.26 (0.81, 6.36)	1.56 (0.81, 6.74)	0.322
Positive ANA	185 (97.9%)	123 (99.2%)	0.652
Positive anti-dsDNA	87 (46.0%)	41 (33.1%)	0.030
Positive anti-RNP	72 (38.1%)	51 (41.1%)	0.675
Positive anti-Sm	53 (28.0%)	37 (29.8%)	0.829
Positive anti-SSA	115 (60.8%)	70 (56.5%)	0.512
Positive anti-SSB	19 (10.1%)	18 (14.5%)	0.309
AI	7.00 (5.00, 9.00)	7.00 (5.00, 9.00)	0.806
CI	2.00 (1.00, 3.00)	2.00 (1.00, 3.00)	0.922
Extrarenal manifestations			
Fever	49 (25.9%)	22 (17.7%)	0.120
Arthralgia	47 (24.9%)	45 (36.3%)	0.041
Photosensitivity	16 (8.5%)	14 (11.3%)	0.526
Rash	16 (8.5%)	14 (11.3%)	0.349
Hypertension	54 (28.6%)	28 (22.6%)	0.295
Alopecia	51 (27.0%)	45 (36.3%)	0.105
Oral ulcers	50 (26.5%)	45 (36.3%)	0.084
First-line treatment			
** Induction therapy**			0.344
Glucocorticoid only	10 (5.3%)	9 (7.3%)	
Immunosuppressive agent pulse	175 (92.6%)	109 (87.9%)	
Novel biological agents pulse	4 (2.1%)	5 (4.0%)	
Other	0 (0.0%)	1 (0.8%)	
Follow-up parameters			
Follow-up (months)	42.6 (16.9, 83.4)	44.0 (17.0, 82.7)	0.713
Endpoint	44 (23.3%)	29 (23.4%)	0.983

Values are presented as the mean ± standard deviation, median (interquartile range) or n (%). LN, lupus nephritis; SLE, systemic lupus erythematosus; MAP, mean arterial pressure; BMI, body mass index; UTP, urinary total protein; WBC, white blood cell; BASO%, basophil percentage; RBC, red blood cell; Hb, hemoglobin; HCT, haematocrit; PLT, platelet; UA, uric acid; eGFR, estimated glomerular filtration rate; hs.CRP, hypersensitive C-reactive protein; AI, activity index; CI, chronicity index; ANA, antinuclear antibodies; dsDNA, anti-double stranded deoxyribonucleic antibody; anti-Sm, anti-Smith antibody; anti-RNP, anti-ribosomal P antibody; anti-SSA, anti-SSA antibody; anti-SSB, anti-SSB antibody.

a BMI was calculated as weight/height^2^;

b MAP was calculated by diastolic blood pressure + systolic blood pressure/3 based on initial blood pressure measurements upon admission;

c eGFR was calculated by the CKD-EPI (Chronic Kidney Disease Epidemiology Collaboration) equation.

**Figure 3:**
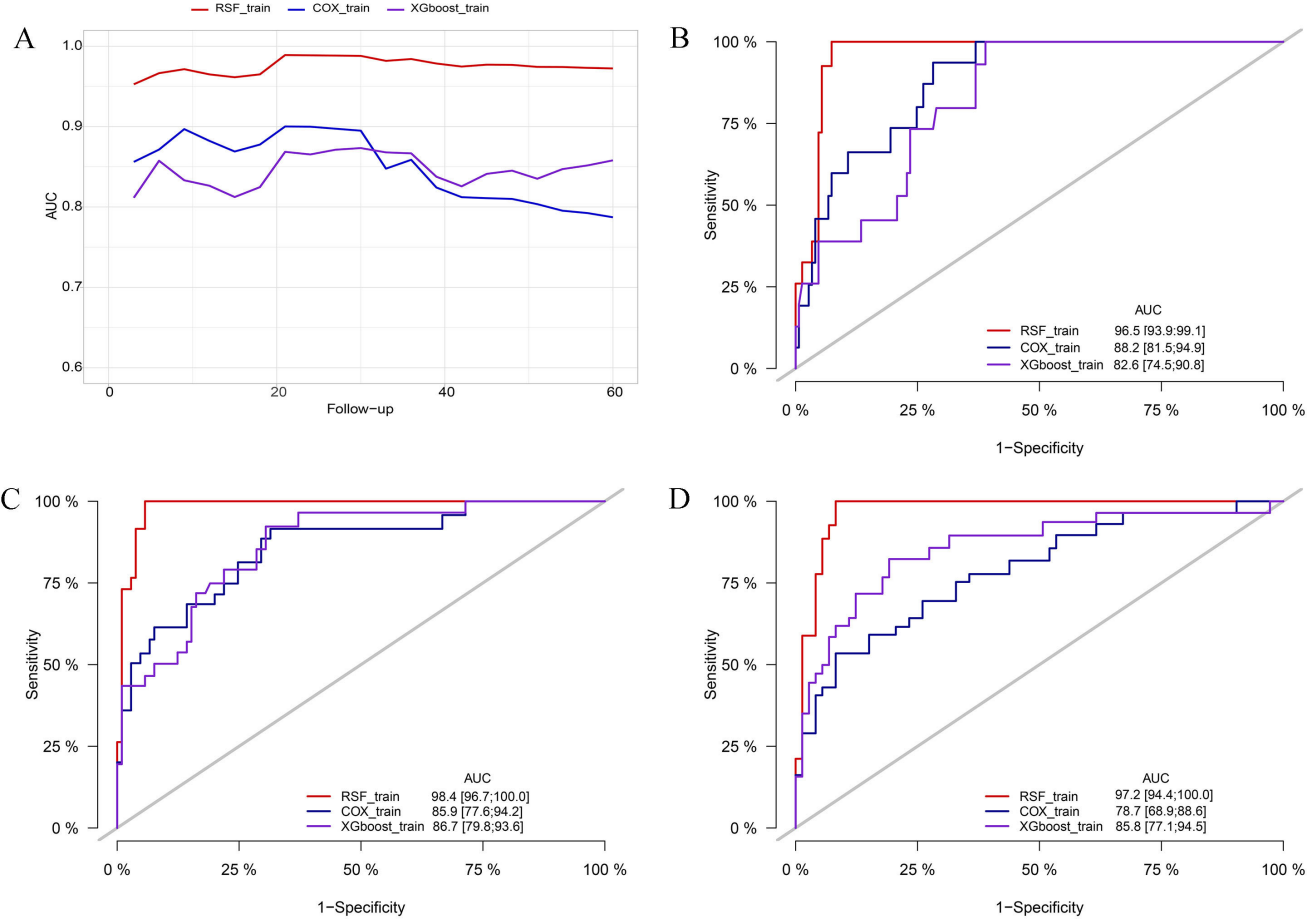
Comparison of AUC among the RSF, XGboost, and Cox model in the training cohort. **(A)** comparison of tAUC among the RSF, XGboost, and Cox model. **(B)** AUC at 12 months. **(C)** AUC at 36 months. **(D)** AUC at 60 months. tAUC, time-independent area under curve; RSF, random survival forest; XGboost, eXtreme Gradient Boosting; Cox, cox regression model; AUC, area under curve.

**Figure 4:**
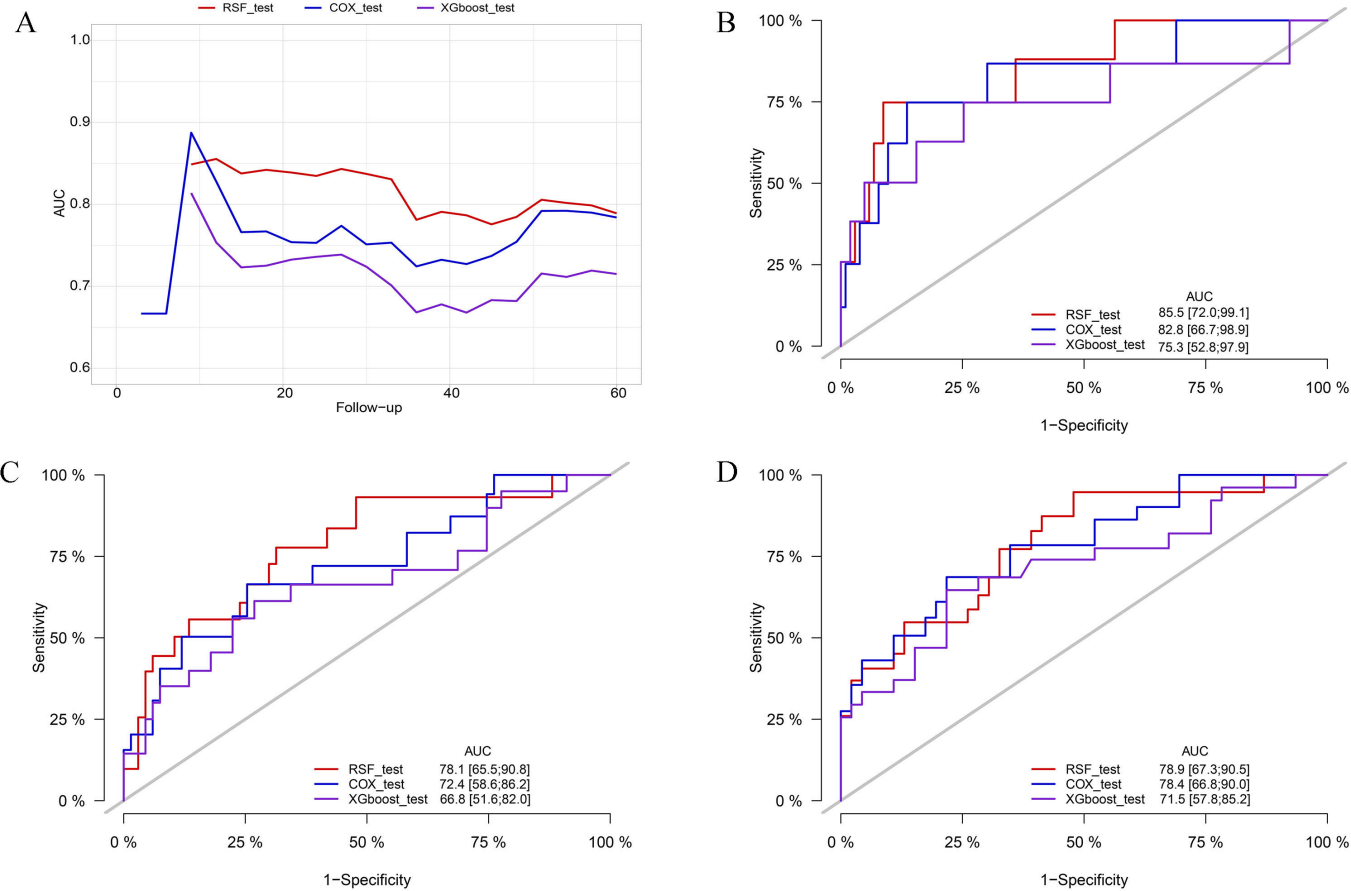
Comparison of AUC among the RSF, XGboost, and Cox model in the testing cohort. **(A)** comparison of tAUC among the RSF, XGboost, and Cox model. **(B)** AUC at 12 months. **(C)** AUC at 36 months. **(D)** AUC at 60 months. tAUC, time-independent area under curve; RSF, random survival forest; XGboost, eXtreme Gradient Boosting; Cox, cox regression model; AUC, area under curve.

In the training cohort, the RSF model demonstrated superior discrimination with AUCs of 0.965 (0.939, 0.991), 0.984 (0.967, 1.00) and 0.972 (0.944, 1.00) at 12-, 36-, and 60-months, respectively. The XGboost model with AUCs of 0.826 (0.745, 0.908), 0.867 (0.798, 0.936) and 0.858 (0.771, 0.945), and Cox model with AUCs of 0.882 (0.815, 0.949), 0.859 (0.776, 0.942), 0.787 (0.689, 0.886) at the same time intervals (**Figure 3**). In the testing set, the RSF model also demonstrated great discrimination with an AUC of 0.855 (0.720, 0.991), 0.781 (0.665, 0.908), and 0.789 (0.673, 0.905) at 12-, 36- and 60-months. By contrast, the AUCs for the XGboost model at the same time intervals were 0.753 (0.528, 0.979), 0.668 (0.516, 0.820), and 0.715 (0.578, 0.852) as well as the Cox model were 0.828 (0.667, 0.989), 0.724 (0.586, 0.862), and 0.784 (0.668, 0.900), respectively (**Figure 4**). Moreover, the RSF model had the higher C-index in both the training [0.950 (0.928, 0.965) vs 0.790 (0.707, 0.847) vs 0.803 (0.723, 0.860), *P* < 0.001] and testing set [0.771 (0.667, 0.848) vs 0.683 (0.543, 0.784) vs 0.727 (0.615, 0.809), *P* < 0.001] (**Table 3**).

**Table 3:** The C-index and IBS score in the training and testing cohort.

Prediction models	Training cohort		Testing cohort	
	C-index (95%CI)	IBS (95%CI)	C-index (95%CI)	IBS (95%CI)
RSF model	0.950 (0.928, 0.965)	0.086 (0.070, 0.104)	0.771 (0.667, 0.848)	0.144 (0.132, 0.154)
XGboost model	0.790 (0.707, 0.847)	0.124 (0.108, 0.140)	0.683 (0.543, 0.784)	0.146 (0.139, 0.160)
Cox model	0.803 (0.723, 0.860)	0.125 (0.117, 0.131)	0.727 (0.615, 0.809)	0.146 (0.132, 0.160)
*P-value*	<0.001	–	<0.001	–

IBS, integrated brier score; random survival forest; Cox, Cox regression; XGboost, eXtreme Gradient Boosting; C-index, concordance index; IBS, integrated brier score.

#### 3.4.2 Comparison of the calibration accuracy of the RSF, XGboost, and Cox model

The IBS was used to assess the calibration of RSF, XGboost, and Cox model. Among them, in the testing cohorts, the RSF model showed the lowest IBS with 0.144 (0.132, 0.154), followed by the XGboost model with an IBS of 0.146 (0.139, 0.160), and the Cox model with an IBS of 0.146 (0.132, 0.160) (**Table 3**). In addition, calibration plots of the RSF model demonstrated that the observed and predicted risks were generally concordant across the entire range of predicted risks, which validated the excellent performance of the RSF model (**Supplementary Figure S3**).

#### 3.4.3 Comparison of prediction efficiency of RSF, XGboost, and Cox model

Compared with the Cox model, the categorical NRI of the RSF model for the 12-, 36-, and 60-months occurrence of combined events were 0.460 (0.137, 0.688), 0.375 (0.146, 0.591) and 0.357 (0.106, 0.557), respectively. For the XGboost model, the NRI values were -0.019 (-0.299, 0.196), -0.053 (-0.266, 0.055) and -0.092 (-0.295, 0.011). The RSF model had an IDI of 0.173 (0.064, 0.268), 0.182 (0.094, 0.264), and 0.158 (0.076, 0.230) at 12-, 36-, and 60-months, respectively. The XGboost model showed an IDI of 0.021 (-0.082, 0.121), -0.017 (-0.102, 0.059) and -0.023 (-0.104, 0.053). These results indicated that the RSF model exhibited superior predictive capability compare to the Cox model (**Table 4**).

**Table 4:** NRI and IDI of prediction models compared with reference model.

Models		Cox model	RSF model	XGboost model	*P* ^a^	*P* ^b^
NRI	12-month	reference	0.460 (0.137, 0.688)	-0.019 (-0.299, 0.196)	0.008	0.603
	36-month		0.375 (0.146, 0.591)	-0.053 (-0.266, 0.055)	0.004	0.635
	60-month		0.357 (0.106, 0.557)	-0.092 (-0.295, 0.011)	0.012	0.328
IDI	12-month	reference	0.173 (0.064, 0.268)	0.021 (-0.082, 0.121)	0.002	0.655
	36-month		0.182 (0.094, 0.264)	-0.017 (-0.102, 0.059)	<0.001	0.635
	60-month		0.158 (0.076, 0.230)	-0.023 (-0.104, 0.053)	<0.001	0.494

NRI, net reclassification improvement; IDI, integrated discrimination improvement; RSF, random survival forest;; XGboost, eXtreme Gradient boosting.

*P ^a^* was calculated by comparing RSF model with the Cox model.

*P ^b^* was calculated by comparing XGboost model with Cox model.

#### 3.4.4 Decision curve analysis of RSF, XGboost, and Cox model

**Figure 5:**
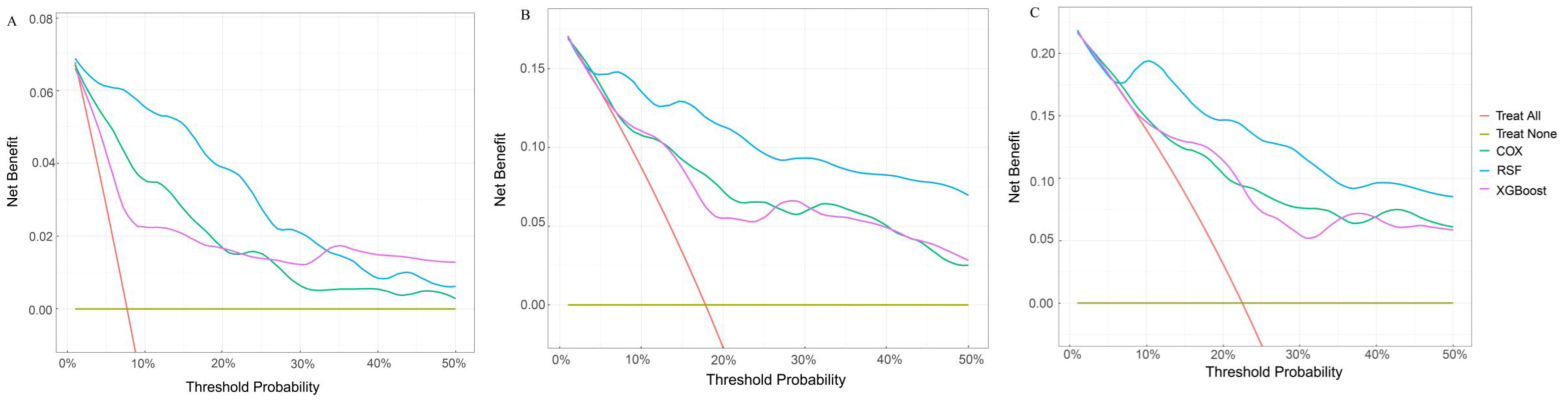
Comparison of DCA plots among the RSF, XGboost, and Cox model. **(A)** DCA plots at 12 months. **(B)** DCA plots at 36 months. **(C)** DCA plots at 60 months. DCA, decision curve analysis. The X-axis is the threshold probability for clinical intervention (range: 0-50%). Y-axis is the net benefit. The red line represents “treat all” that means the assumption that all patients receive intervention. The yellow line represents “treat none” representing the assumption that no patients receive intervention. The green line represents the Cox model. The blue line represents the RSF model. The purple line represents the XGBoost model. DCA, decision curve analysis; RSF, random survival forest; XGboost, eXtreme Gradient Boosting; Cox, cox regression model.

The DCA offers a graphical representation to visualize the practical applicability of each model. As shown in **Figure 5**, the RSF model exhibits a higher net benefit than the XGboost model and Cox model across all threshold probabilities, including the 12-, 36-, and 60-month thresholds. This suggests that the RSF model may offer greater clinical benefit than the XGboost model and Cox model.

### 3.5 Renal outcomes of class IV ± V LN patients under CI scores

**Figure 6:**
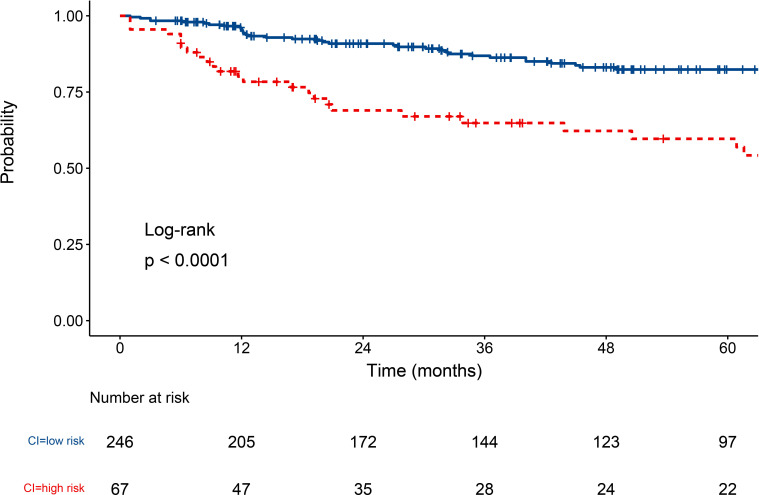
Survival curve of class IV ± V LN patients under CI score. LN, lupus nephritis; CI, chronic index.

CI is the variable with the highest VIMP value following eGFR. To further assess the impact of kidney pathology on the patient renal outcomes, we conducted survival analyses stratified by CI scores. Based on the MSRSM (**Supplementary Figure S4**), the optimal cutoff value for the CI score was determined to be 4. Using this optimal cutoff value, patients with class IV ± V LN were categorized into high and low risk groups, and Kaplan-Meier survival curves revealed that patients with elevated CI scores exhibited a poorer renal prognosis (**Figure 6**).

## 4 Discussion

In this study, we conducted a comparative analysis of the clinical features between patients with class IV and IV+V LN, followed by survival analyses to assess the prognosis. Subsequently, we assessed the critical risk factors influencing the prognosis of class IV ± V LN who underwent renal biopsy over a nearly 10-year period and developed novel prognostic prediction models. Class IV ± V LN patients exhibit distinct pathological features, yet they demonstrate comparable clinical manifestations, treatment strategies, and long-term prognosis. eGFR, CI, age, BASO%, RBC, MAP, and UA were identified as the risk variables. In terms of discriminatory capability and clinical applicability, the RSF model showed superior predictive prowess compared to the XGboost and Cox regression models.

In this study, we hold that there is no difference in prognosis of class IV and IV+V LN patients. Indeed, previous studies comparing the prognosis between pure class IV and mixed class IV+V LN have yielded conflicting results. While some studies supported that class IV+V LN with significantly worse renal outcomes (12, 14, 30), others supported no statistically significant differences in renal survival between class IV and IV+V LN (13, 31). Most studies grouped class III/IV+V as “mixed proliferative” and class III/IV as “pure proliferative”, without isolating comparisons between class IV and IV+V LN. Moreover, small sample sizes or imbalanced proportions of mixed and pure proliferative LN cohorts, which may limit statistical power and result the different conclusion. In our study, we focused on class IV+V and IV LN patients and conducted a comparison of class IV+V and IV LN patients with a balanced cohort ratio (approximately 1:1), which can increase the reliability of the research results. We demonstrated that patients with class IV+V LN were found to be similar to those with class IV in terms of clinical manifestations, treatment strategies, and long-term prognosis, despite having more severe chronic kidney damage. These findings support the advice of the existing guidelines that the mixed proliferative LN should be managed similarly to pure proliferative LN (15). In addition, class IV LN patients tend to exhibit higher levels of albumin and BASO%, as well as lower levels of RBC and hemoglobin, which does not necessarily result in differences in the prognosis of class IV ± V LN patients. Therefore, class IV and class IV+V can be studied together and result in the same clinical management decision in clinical practice.

Our findings indicate that compared to the Cox and XGboost model, the RSF model exhibits strong robustness in predicting the progression of class IV ± V LN patients and provides more precise probability estimates over time. In terms of predictive accuracy, the RSF model demonstrates a higher C-index and AUC values. Additionally, the RSF model exhibits lower IBS, indicating superior calibration ability. Furthermore, the RSF model has higher NRI and IDI values, indicating superior discrimination ability. The RSF algorithm has the ability to mitigate overfitting through two random sampling processes, without the constraints of assumptions such as proportional hazards and log-linear relationships (32, 33). XGboost can train weak classifiers using the negative gradient information of the loss function, effectively preventing overfitting and improving model performance (34). Both XGboost and RSF have strong nonlinear variable processing capabilities (35). In contrast, the Cox model only identifies linear relationships (36). The exceptional performance of RSF can potentially be attributed to the underlying nonlinear relationships between variables and outcomes.

Analyzing the risk factors through the RSF prediction model has the potential to enhance disease management strategies and optimize patient prognosis. CI emerged as the most critical risk factor affecting the prognosis of patients with class IV ± V LN, independent of eGFR. The AI and CI scores of the NIH system are commonly employed as supplementary tools alongside the 2003 ISN/RPS-LN classification system (3). CI assesses the degree of chronicity of glomerular and tubulointerstitial lesions in renal tissue from LN patients in a semi-quantitative manner and has been recognized as a predictor of adverse renal outcome (37–40). In proliferative LN, chronic kidney damage can progress rapidly, even among patients who clinically appear to respond favorably to aggressive immunosuppressive therapy (41). Therefore, clinicians should pay greater attention to the CI score and tailor the therapeutic approach to the degree of chronic injury in order to preserve renal function and improve long-term prognosis. Furthermore, when stratifying risks based on CI scores, physicians can assess the survival prospects of patients, especially those in the high-risk group (CI > 4).

Age has been shown to be a primary factor associated with mortality in LN but not with ESRD (39, 42, 43). Notably, age emerged as an independent predictor of adverse prognosis in the class IV ± V LN patients, which may be attribute to the inclusion of mortality in the composite endpoints in this study.

BASO% was identified as a clinical predictor for the prognosis of class IV ± V patients in this study. BASO has been shown to be important contributors to the pathogenesis of SLE, particularly in the development of LN. During the pathogenesis of LN, BASO has the capability to intensify the production of autoantibodies, amplify the formation of immune complexes, and ultimately resulting in the deposition of these complexes within the kidney (44). Liang et al. observed that active LN often exhibits lower levels of circulating BASO counts, demonstrating an inverse relationship with pathological activity (45). Consequently, variations in BASO levels not only as a reliable indicator of LN activity, but also potentially offer pivotal clues for anticipating the long-term prognostic outcomes of LN.

MAP, RBC, and UA were similarly identified as predictors for the adverse outcome of class IV ± V LN patients in this study. Hypertension stands as a significant contributor to unfavorable renal outcomes in LN patients, and its presence heightens renal histologic activity and accelerates renal dysfunction (46–48). Therefore, blood pressure control is of significant importance for the long-term prognosis management of patients. Anemia has also been identified being associated with adverse outcome in LN (12, 49, 50). Tubulointerstitial lesions lead to reduced erythropoietin production, thereby decreasing RBC counts, which have been strongly correlated with renal prognosis (12, 51). Consequently, the RBC count has the potential to be a vital prognostic indicator for class IV ± V LN. Recently, it has been established that elevated serum UA (SUA) levels serve as an independent risk factor and predictor of unfavorable long-term outcomes among patients with LN, aligning with the results of this study (52–54). With each 100 μmol/L increase in SUA levels, the risk of ESRD or death increased by 10% (54). Therefore, monitoring SUA levels and early intervention have the potential to improve long-term outcomes in LN patients, especially those with class IV ± V LN.

Our RSF model offers several advantages over previous studies, which rely primarily on clinical and pathological factors to predict outcomes. These advantages include a more precise target population, broader background data, and more targeted outcomes. However, there are also some limitations to this study. Firstly, the relatively small sample size and the absence of external validation are the main constraints. To address these limitations, we plan to conduct a larger prospective study, enrolling a broader cohort of eligible patients to further validate our model. Additionally, as the patients included in this study were from China, further verification is required to assess the generalizability of our prediction model to other ethnic groups. Finally, our data collection was limited to basic information on treatment strategies and was incapable of evaluating the impact of treatment during the follow-up period. Despite treatment not being a prognostic variable in our study, additional investigations examining the impact of treatment may be warranted. Despite these limitations in this study, we maintain that our findings can provide valuable insights for initiating early therapeutic intervention and optimizing the long-term prognosis for patients suffering from class IV ± V LN.

## 5 Conclusion

Class IV ± V LN exhibit distinct pathological features, yet demonstrate comparable clinical manifestations, treatment strategies, and long-term prognosis. Therefore, this study is the first to identify the key risk factors (eGFR, CI, age, BASO%, RBC, MAP, and UA) that influence class IV ± V LN, and to establish ML models including the RSF and XGboost model, to compare with the Cox model. The RSF model exhibits superior survival prediction and provides more precise prognostic stratification. The implications of these findings have the potential to enhance clinical decision-making within medical practice, thus underscoring the necessity for rigorously designed studies to provide further validation.

## Data Availability

The datasets presented in this article are not readily available because The dataset contains personal health records, etc.], and is therefore subject to strict privacy and ethical restrictions. Access to the dataset is limited to authorized researchers who have obtained approval from ethics committee and comply with all relevant data protection regulations. Requests to access the datasets should be directed to sunshiren@medmail.com.cn.

## Supplementary material

Supplementary Figure 1 Parameter of developing RSF model. RSF, random survival forests.

Supplementary Figure 2 Survival curve of class IV and IV+V LN patients. LN, lupus nephritis.

Supplementary Figure 3 The calibration curve of the RSF model in the training cohorts and testing cohorts **(A)** calibration curve at 12 months. **(B)** calibration curve at 36 months. **(C)** calibration curve at 60 months. RSF, random survival forests. The red line represents the performance of RSF model in the training set with the blue line shows its error bars. The yellow line represents the performance of RSF model in the testing set with the green line shows its error bars.

Supplementary Figure 4 The optimal cutoff value for CI by the MSRSM. CI, chronic index. MSRSM, Maximally Selected Rank Statistics Method.
