# Supplementary material for: Clinical characteristics, prognosis, and predictive modeling in class IV ± V lupus nephritis
Source: Front Immunol. 2025 May 15;16:1580146. doi: 10.3389/fimmu.2025.1580146 (PMC12119475; doi:10.3389/fimmu.2025.1580146)
Supplement: Supplementary Figure 1 — Parameter of developing RSF model. RSF, random survival forests. [file DataSheet1.docx]

**Supplemental Materials**

**Table S1. TRIPOD Checklist.**

**Table S2. All variables included in the analysis.**

**Table S3. The 95% CI of bootstrapped VIMP.**

**Table S4. Multivariate Cox regression analysis of key factors affecting renal combined events in class IV±V LN patients.**

**Figure S1. Parameter of developing RSF model.**

**Figure S2. Survival curve of class IV and IV+V LN patients.**

**Figure S3. The calibration curve of the RSF model in the training cohorts and testing cohorts.**

**Figure S4. The optimal cutoff value for CI by the MSRSM.**

| **Table S1. TRIPOD Checklist.** | | | | |
| --- | --- | --- | --- | --- |
| **Section/Topic** | **Item** |  | **Checklist Item** | **Page** |
| **Title and abstract** | | | | |
| Title | 1 | D;V | Identify the study as developing and/or validating a multivariable prediction model, the target population, and the outcome to be predicted. | 1 |
| Abstract | 2 | D;V | Provide a summary of objectives, study design, setting, participants, sample size, predictors, outcome, statistical analysis, results, and conclusions. | 2 |
| **Introduction** | | | | |
| Background and objectives | 3a | D;V | Explain the medical context (including whether diagnostic or prognostic) and rationale for developing or validating the multivariable prediction model, including references to existing models. | 3-4 |
|  | 3b | D;V | Specify the objectives, including whether the study describes the development or validation of the model or both. | 3-4 |
| **Methods** | | | | |
| Source of data | 4a | D;V | Describe the study design or source of data (e.g., randomized trial, cohort, or registry data), separately for the development and validation data sets, if applicable. | 4 |
|  | 4b | D;V | Specify the key study dates, including start of accrual; end of accrual; and, if applicable, end of follow-up. | 4 |
| Participants | 5a | D;V | Specify key elements of the study setting (e.g., primary care, secondary care, general population) including number and location of centres. | 4 |
|  | 5b | D;V | Describe eligibility criteria for participants. | 4 |
|  | 5c | D;V | Give details of treatments received, if relevant. | 5 |
| Outcome | 6a | D;V | Clearly define the outcome that is predicted by the prediction model, including how and when assessed. | 5 |
|  | 6b | D;V | Report any actions to blind assessment of the outcome to be predicted. | 5 |
| Predictors | 7a | D;V | Clearly define all predictors used in developing or validating the multivariable prediction model, including how and when they were measured. | 5-6 |
|  | 7b | D;V | Report any actions to blind assessment of predictors for the outcome and other predictors. | 5-6 |
| Sample size | 8 | D;V | Explain how the study size was arrived at. | 5 |
| Missing data | 9 | D;V | Describe how missing data were handled (e.g., complete-case analysis, single imputation, multiple imputation) with details of any imputation method. | 6 |
| Statistical analysis methods | 10a | D | Describe how predictors were handled in the analyses. | 5-6 |
|  | 10b | D | Specify type of model, all model-building procedures (including any predictor selection), and method for internal validation. | 5-6 |
|  | 10c | V | For validation, describe how the predictions were calculated. | 6 |
|  | 10d | D;V | Specify all measures used to assess model performance and, if relevant, to compare multiple models. | 6 |
|  | 10e | V | Describe any model updating (e.g., recalibration) arising from the validation, if done. | 6-7 |
| Risk groups | 11 | D;V | Provide details on how risk groups were created, if done. | None |
| Development vs. validation | 12 | V | For validation, identify any differences from the development data in setting, eligibility criteria, outcome, and predictors. | 6-7 |
| **Results** | | | | |
| Participants | 13a | D;V | Describe the flow of participants through the study, including the number of participants with and without the outcome and, if applicable, a summary of the follow-up time. A diagram may be helpful. | 7 (Figure 1) |
|  | 13b | D;V | Describe the characteristics of the participants (basic demographics, clinical features, available predictors), including the number of participants with missing data for predictors and outcome. | 7(Table1, Table S2, Table S3, Figure S2) |
|  | 13c | V | For validation, show a comparison with the development data of the distribution of important variables (demographics, predictors and outcome). | 7 (Figure1) |
| Model development | 14a | D | Specify the number of participants and outcome events in each analysis. | 7 |
|  | 14b | D | If done, report the unadjusted association between each candidate predictor and outcome. | 8 |
| Model specification | 15a | D | Present the full prediction model to allow predictions for individuals (i.e., all regression coefficients, and model intercept or baseline survival at a given time point). | 7-8 |
|  | 15b | D | Explain how to the use the prediction model. | 7-8 |
| Model performance | 16 | D;V | Report performance measures (with CIs) for the prediction model. | 7-9 (Figure 3; Table 2; Figure S2) |
| Model-updating | 17 | V | If done, report the results from any model updating (i.e., model specification, model performance). | None |
| **Discussion** | | | | |
| Limitations | 18 | D;V | Discuss any limitations of the study (such as nonrepresentative sample, few events per predictor, missing data). | 12 |
| Interpretation | 19a | V | For validation, discuss the results with reference to performance in the development data, and any other validation data. | 9-12 |
|  | 19b | D;V | Give an overall interpretation of the results, considering objectives, limitations, results from similar studies, and other relevant evidence. | 9-12 |
| Implications | 20 | D;V | Discuss the potential clinical use of the model and implications for future research. | 12 |
| **Other information** | | | | |
| Supplementary information | 21 | D;V | Provide information about the availability of supplementary resources, such as study protocol, Web calculator, and data sets. | 13-14 |
| Funding | 22 | D;V | Give the source of funding and the role of the funders for the present study. | 15 |

| \| **Table S2. All variables included in the analysis.** \| \| \| --- \| --- \| \| **Demographic characteristics** \| \| \| Age \| Temperature \| \| Gender \| Respirations \| \| Duration of SLE \| Temperature \| \| Body mass index \| Pulse \| \| **Clinical complications** \| \| \| Fever \| Alopecia \| \| Rash \| Oral ulcers \| \| Arthralgia \| Hypertension \| \| Photosensitivity \|  \| \| **Clinical characteristics** \| \| \| Mean arterial pressure \| Alkaline phosphatase \| \| Urine total protein \| Cholesterol \| \| White blood cell count \| Triglyceride \| \| Neutrophilic granulocyte percentage \| High-density lipoprotein \| \| Lymphocyte ratio \| Low-density lipoprotein \| \| Monocyte percentage \| Serum glucose \| \| Eosinophil percentage \| Serum K \| \| Basophil percentage \| Serum Na \| \| Red blood cell count \| Serum CL \| \| Hemoglobin \| Serum Ca \| \| Hematocrit \| CO_2_CP \| \| Mean corpuscular volume \| Fibrinogen degradation products \| \| Mean corpusular hemoglobin \| Prothrombin time \| \| CV (Mean corpusular hemoglobin concerntration percentage) \| Activated partial thromboplastin time \| \| CV (red blood cell distribution width percentage) \| Fibrinogen \| \| SD (red blood cell distribution width percentage) \| Thrombin time \| \| Platelets \| D-dimer \| \| Platelet distribution width \| Prothrombin time ratio \| \| Mean platelet volume \| International normalized ratio \| \| Urine specific gravity \| Serum IgG \| \| Urinary pH \| Serum IgA \| \| Urinary red blood cell \| Serum IgM \| \| Urinary epithelial cell quantification \| Serum tIgE \| \| Urinary leukocyte \| Serum C3 \| \| Urinary proteinuria \| Serum C4 \| \| Urinary CAST \| Serum C1NIH \| \| Urinary Bacteria CAST \| Antihemolytic streptococcin o \| \| Urinary Pathological CAST \| Rheumatoid factor \| \| Blood urea nitrogen \| Hypersensitive C-reactive protein \| \| Estimated glomerular filtration rate \| Positive antinuclear antibodies \| \| Serum uric acid \| Positive anti-dsDNA \| \| Cystatin C \| Positive anti-RNP \| \| Alanine amino transferase \| Positive anti-Sm \| \| Aspartate transaminase \| Positive anti-SSA \| \| Total protein \| Positive anti-SSB \| \| Serum albumin \| Positive anti-Ro.52 \| \| Total bilirubin \| Positive anti-Scl.70 \| \| Indirect bilirubin \| Positive anti-Jo.1 \| \| **Renal pathological changes** \| \| \| Activity index \| Chronic index \| \| Mesangial deposition of IgG \| Mesangial deposition of IgA \| \| Mesangial deposition of IgM \| Mesangial deposition of C3 \| \| Mesangial deposition of C4 \| Mesangial deposition of C1Q \| \| Mesangial deposition of FiB \|  \| \| **Treatment options** \| \| \| Induction therapy \| Glucocorticoid \| \| Immunosuppressive agent \| Mycophenolate mofetil \| \| Cyclosporin \| Tacrolimus \| \| Hydroxychloroquine \| Rituximab \| \| Belimumab \| Research Analyst Assessment Specialist \| \| Adrenergic receptor binder \| Angiotensin converting enzyme inhibitors \| \| Calcium channel blocker \|  \| |
| --- | --- | --- | --- | --- | --- | --- | --- | --- | --- | --- | --- | --- | --- | --- | --- | --- | --- | --- | --- | --- | --- | --- | --- | --- | --- | --- | --- | --- | --- | --- | --- | --- | --- | --- | --- | --- | --- | --- | --- | --- | --- | --- | --- | --- | --- | --- | --- | --- | --- | --- | --- | --- | --- | --- | --- | --- | --- | --- | --- | --- | --- | --- | --- | --- | --- | --- | --- | --- | --- | --- | --- | --- | --- | --- | --- | --- | --- | --- | --- | --- | --- | --- | --- | --- | --- | --- | --- | --- | --- | --- | --- | --- | --- | --- | --- | --- | --- | --- | --- | --- | --- | --- | --- | --- | --- | --- | --- | --- | --- | --- | --- | --- | --- | --- | --- | --- | --- | --- | --- | --- | --- | --- | --- | --- | --- | --- | --- | --- |

| **Table S3. The 95% CI of bootstrapped VIMP.** | | | | |
| --- | --- | --- | --- | --- |
| **Variables** | **lower** | **mean** | **upper** | ***p-value*** |
| eGFR | 0.044 | 0.096 | 0.149 | 0.000169 |
| CI | 0.032 | 0.094 | 0.157 | 0.001473 |
| Age | 0.009 | 0.042 | 0.076 | 0.00643 |
| CO_2_CP | 0.009 | 0.033 | 0.058 | 0.003994 |
| MAP | 0.005 | 0.027 | 0.049 | 0.008297 |
| EO% | 0.004 | 0.026 | 0.048 | 0.011063 |
| RBC | 0.006 | 0.023 | 0.004 | 0.004517 |
| UA | 0.005 | 0.023 | 0.041 | 0.006138 |
| AI | 0.002 | 0.002 | 0.042 | 0.015839 |
| BASO% | 0.001 | 0.021 | 0.042 | 0.020745 |
| hs.CRP | 0.003 | 0.020 | 0.037 | 0.009129 |
| Note: 95% CI, 95% confidence interval; VIMP, variable importance. eGFR, estimated glomerular filtration rate. CI, chronicity index. MAP, mean arterial pressure. EO%, percentage of eosinophil. RBC, red blood cell. UA, uric acid. AI, activity index. BASO%, percentage of basophilic granulocyte. hs.CRP, hypersensitive C-reactive protein. | | | | |

**Table S4.** **Multivariate Cox regression analysis of key factors affecting renal combined events in class IV±V LN patients.**

**.**

| **Variables** | **Univariate Cox analysis** | | **Multivariate Cox analysis** | | |  |
| --- | --- | --- | --- | --- | --- | --- |
|  | **HR (95% CI)** | ***P*** | **HR (95% CI)** | ***P*** | |  |
| Age | 1.03 (1.01-1.05) | 0.001 | 1.03 (1.01-1.05) | | 0.009 |  |
| MAP | 1.03 (1.02-1.04) | < 0.001 | Not included | |  |  |
| BASO% | 4.73e+46 (1.27e+46-1.76e+77) | 0.003 | 1.56e+51 (6.93e+15-3.51e+86) | | 0.005 |  |
| RBC | 0.58 (0.43-0.79) | < 0.001 | 0.65 (0.45-0.94) | | 0.023 |  |
| CV(RDW) | 105.88 (9.83-1139.93) | < 0.001 | Not included | |  |  |
| BACT | 1 (1-1) | 0.028 | Not included | |  |  |
| eGFR | 0.98 (0.97-0.99) | < 0.001 | 1.01 (1.00-1.02) | | 0.018 |  |
| UA | 1 (1-1) | < 0.001 | 1.004 (1.002-1.006) | | < 0.001 |  |
| CO_2_CP | 0.91 (0.85-0.97) | 0.006 | Not included | |  |  |
| APTT | 1.02 (1-1.03) | 0.012 | Not included | |  |  |
| TT | 1.02 (1.01-1.04) | 0.006 | Not included | |  |  |
| Pt% | 0.98 (0.97-1) | 0.032 | 0.98(0.96-1.00) | | 0.048 |  |
| hs.CRP | 1.02 (1-1.03) | 0.009 | Not included | | 0.048 |  |
| CI | 1.35 (1.24-1.48) | < 0.001 | 1.23 (1.08-1.40) | | 0.002 |  |
| IGA | 0.44 (0.27-0.72) | 0.001 | Not included | |  |  |
| Hypertension | 2.59 (1.62-4.12) | < 0.001 | Not included | |  |  |
| Hydroxychloroquine | 0.55 (0.34-0.88) | 0.014 | Not included | |  |  |
| CCB | 2.28 (1.43-3.62) | < 0.001 | Not included | |  |  |
| Note: LN, lupus nephritis. HR, Hazard Ratio. 95% CI, 95% confidence interval; MAP, mean arterial pressure. RBC, red blood cell. CV(RDW), CV (red blood cell distribution width percentage). BACT, urinary Bacteria CAST. eGFR, estimated glomerular filtration rate. UA, uric acid. APTT, activated partial thromboplastin time. TT, thrombin time. Pt%, prothrombin time ratio. hs.CRP, hypersensitive C-reactive protein. CI, chronicity index. IGA, mesangial deposition of IgA. CCB, calcium channel blocker. | | | | | | |

**Figure S1. Parameter of developing RSF model.**


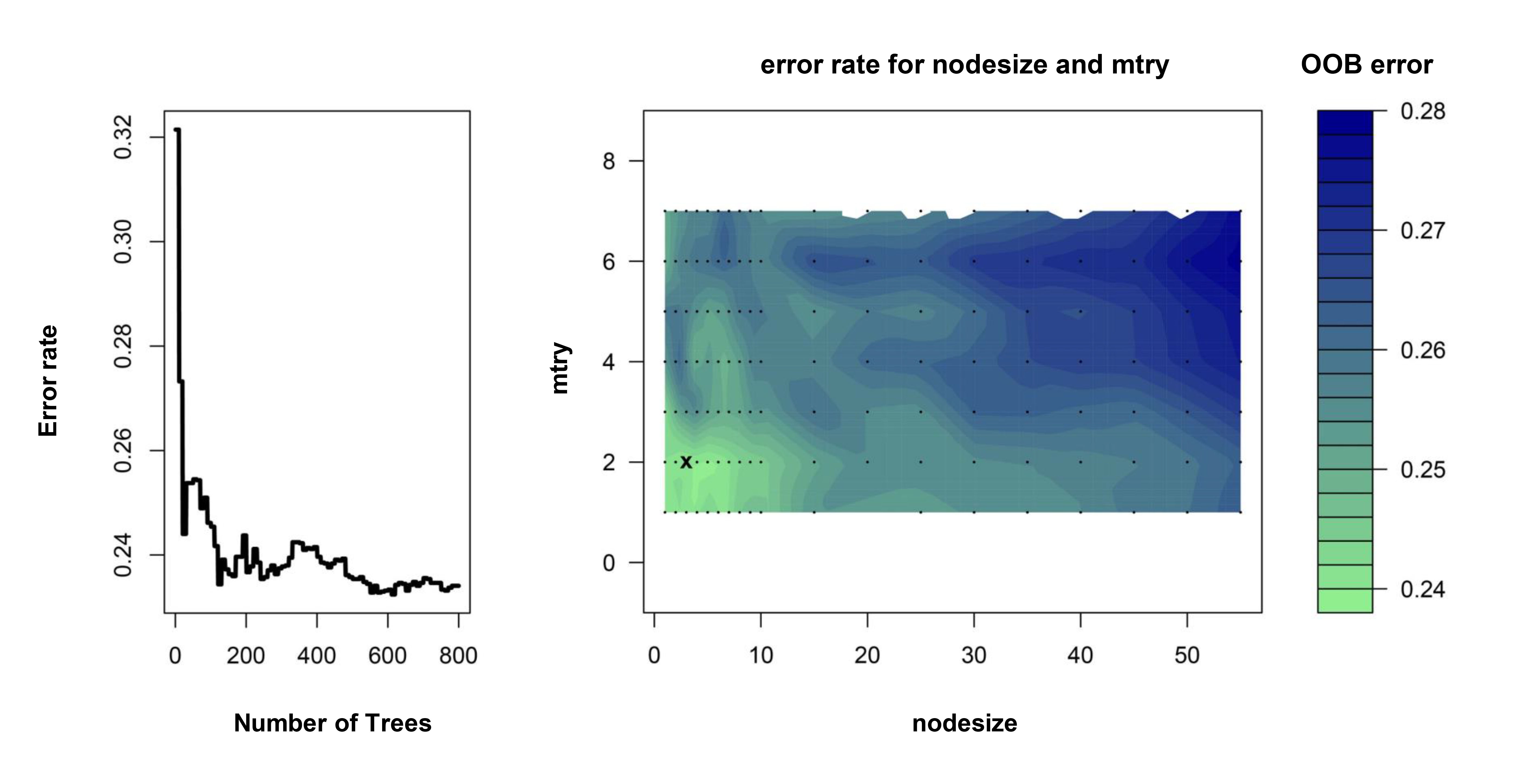


The ntree, the mtry and nodesize was 800, 2 and 3, respectively. RSF, random survival forests.

**Figure S2. Survival curve of class IV and IV+V LN patients.**


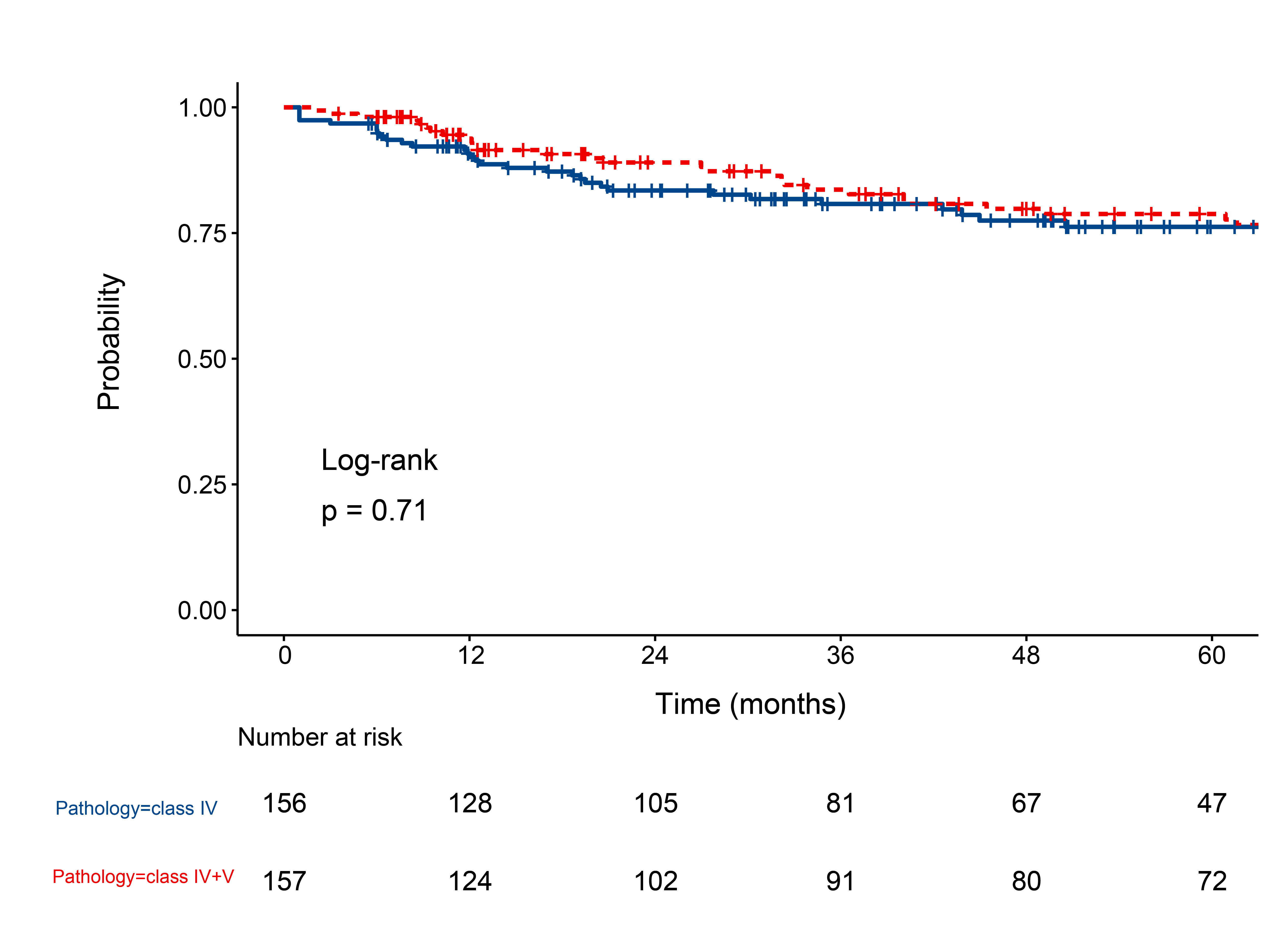


LN, lupus nephritis.

**Figure S3. The calibration curve of the RSF model in the training cohorts and testing cohorts.**


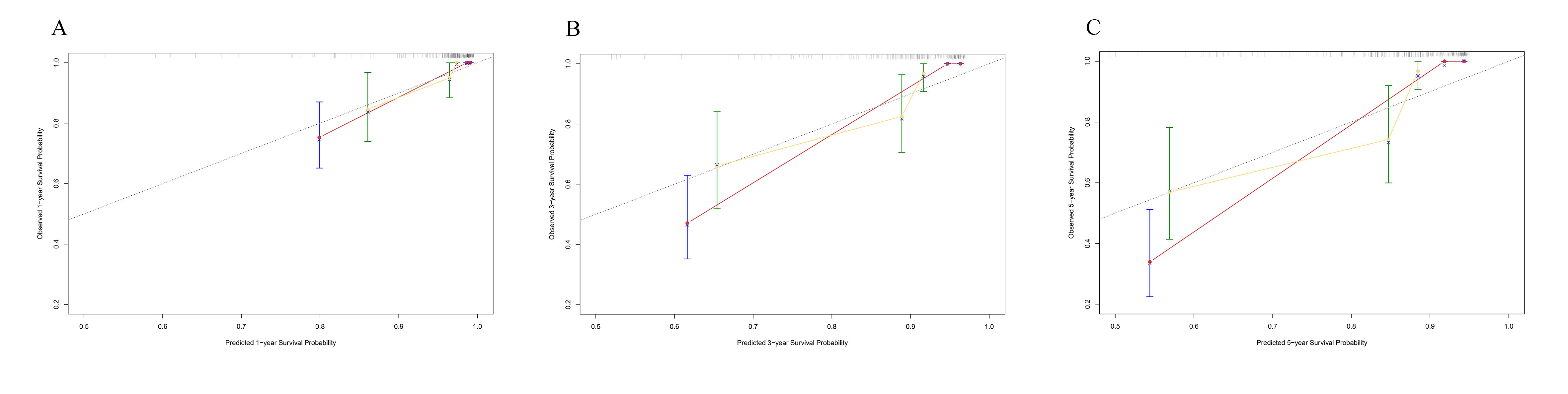


(A) calibration curve at 12 months. (B) calibration curve at 36 months. (C) calibration curve at 60 months. RSF, random survival forests. The red line represents the performance of RSF model in the training set with the blue line shows its error bars. The yellow line represents the performance of RSF model in the testing set with the green line shows its error bars.

**Figure S4. The optimal cutoff value for CI by the MSRSM.**


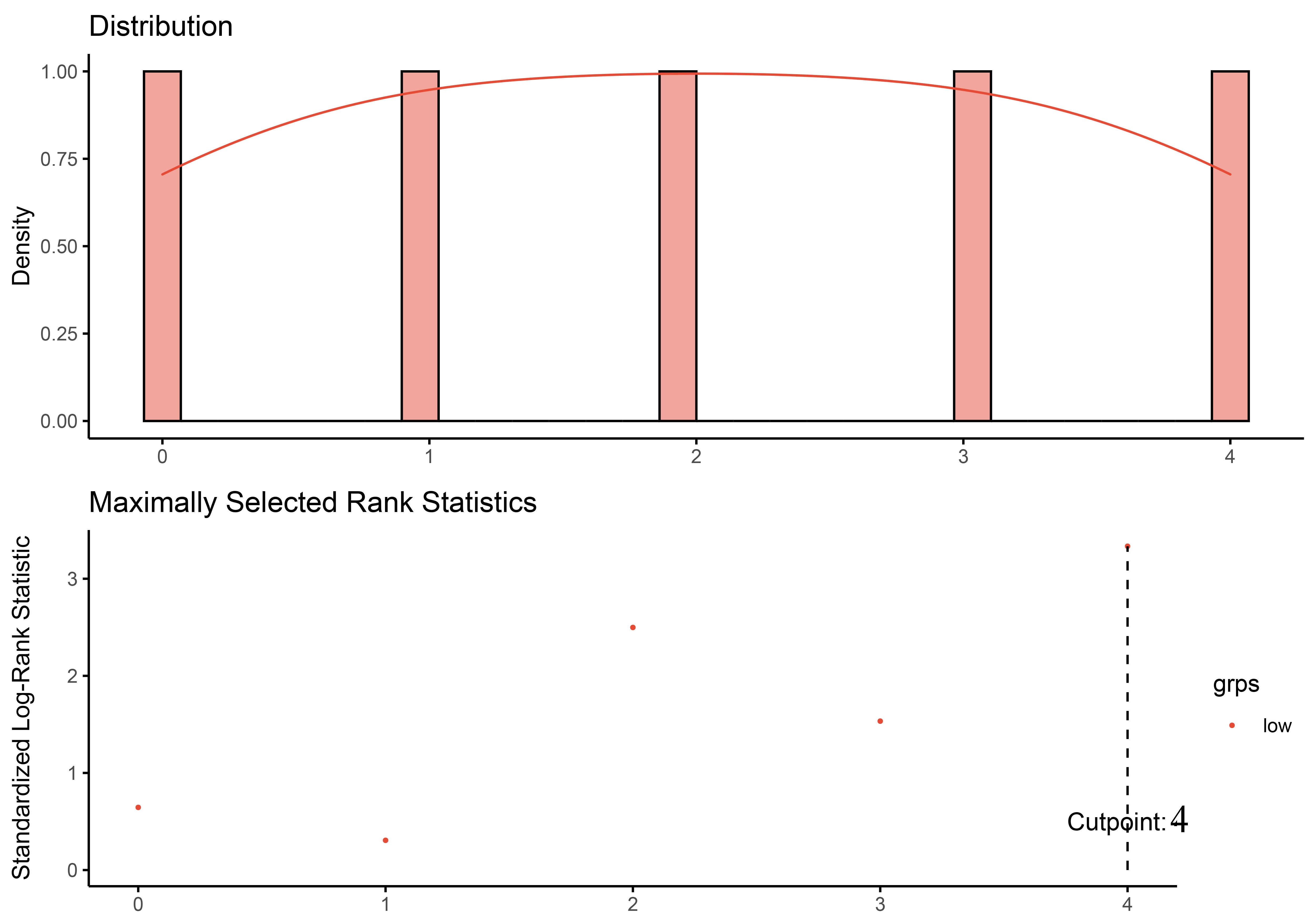


CI, chronic index. MSRSM, Maximally Selected Rank Statistics Method.
